# Kinetics of Expansion of Human Limbal Epithelial Progenitor Cells in Primary Culture of Explants Without Feeders

**DOI:** 10.1371/journal.pone.0081965

**Published:** 2013-12-03

**Authors:** Djida Ghoubay-Benallaoua, Otman Sandali, Pablo Goldschmidt, Vincent Borderie

**Affiliations:** 1 Institut de la Vision, UPMC Université Paris 06, UMR_S 968 / INSERM, U968 / CHNO des XV-XX / CNRS, UMR_7210, Paris, France; 2 Laboratoire du Centre Hospitalier National d’Ophtalmologie des Quinze-Vingts, Paris, France; 3 Banque de Tissus, Établissement Français du Sang, Site Saint-Antoine, Paris, France; Eye Hospital, Charité, Germany

## Abstract

The aims of this study were to determine whether human limbal explant cultures without feeder cells result in expansion of epithelial progenitors and to estimate the optimal expansion time for progenitor cells. Limbal explants from ten human corneas were cultured for 7, 9, 11, 14, 18, and 21 days. Limbal explants from two corneas were enzymatically dissociated or directly cultured for 14 days. Progenitor cells were characterized by their ability to form colonies, by immunocytochemistry, and by quantitative real-time polymerase chain reaction. Colonies were identified after 9, 11, 14, and 18 days of culture, but not after 21 days. The number of colonies per explant was significantly higher after 14 days than after 9 and 21 days. The mean percentage of seeded cells giving rise to clones was 4.03% after 14 days of culture and 0.36% for non-cultured dissociated limbal epithelial cells. The number of cells giving rise to clones per cornea significantly increased from an average of 2275 for non-cultured cells to 24266 for cells cultured for 14 days. Immunocytochemical analysis detected positive staining for cytokeratin (CK) 3, CK5/6/8/10/13/18, CK19, vimentin, p63, and p63α, in both cultures and clones. CK3 expression increased significantly with culture time. Transcript expression was observed for CK3, CK19, vimentin, and Delta N p63α at each culture time point, both in cultures and clones. The optimal culture time for limbal explants in cholera toxin-free Green medium without feeder cells was 14 days leading to the expansion of progenitors.

## Introduction

The ocular surface is covered with three non-keratinizing epithelia: the transparent corneal epithelium, the conjunctival epithelium, and the limbal epithelium overlying the limbal region which lies between the cornea and the sclera [1]. Renewal of the corneal epithelium from the limbal epithelial structures is essential for maintaining the optical properties of the cornea [2].

In patients with limbal stem cell (SC) deficiency, one of the emerging surgical strategies for restoring the corneal epithelial surface is the transplantation of ex vivo expanded limbal epithelial SCs [3-6]. This therapeutic approach involves harvesting of small limbal biopsies from either the patient’s contralateral healthy eye or a donor eye, followed by cell-expansion to produce an epithelial sheet on a transplantable carrier such as fibrin or human amniotic membrane [6-12].

Epithelial cells obtained from the limbus and subsequently cultured in vitro have been shown to be reprogrammable to pluripotency through a simple manipulation of the cell microenvironment [13]. Additionally, the stem cell niche of the limbal epithelial cells can be affected by the culture conditions, including murine 3T3 feeder cells, human amniotic membrane (AM), fibrin, and tissue-culture treated plastic. Limbal explant culture may mimic the limbal progenitor cell niche by preserving in culture the various cells present in the limbal stroma close to the basal epithelial cells. These stromal cells have been shown to favor the maintenance of stemness in culture [14]. The phenotypic characterization of the putative limbal stem cells revealed, by semi quantitative immunohistochemical staining, EGF receptor, integrin α9, p63α, Delta-N p63α, integrin β1, ATP-binding cassette, subfamily G, member 2 (ABCG2), Bmi-1, C/EBPδ, and nestin as possible positive markers, and keratin 3/12, E-cadherin, involucrin, connexin 43, and Hoechst 33342 as possible negative markers for limbal progenitor cells [15-27]. For long-term restoration of damaged ocular surface, preservation of limbal SCs during the culture process and after grafting is needed [3,28]. Furthermore, the success rate after transplantation of autologous cultured limbal epithelial cells depends on the presence of p63+ cells in culture [28]. Whereas the presence of progenitors in limbal epithelial cell cultures has been demonstrated through expression of several markers and colony formation assays, little is known about the rate and kinetics of progenitor cell expansion [30-32].

In order to provide an optimized culture condition supporting preferential expansion and maintenance of the limbal progenitors for therapeutic applications, the present study aimed to assess the expansion of limbal epithelial progenitors in culture and its kinetics according to the clonal growth and preservation of progenitor phenotype. 

## Methods

This study was carried out according to the tenets of the Declaration of Helsinki and it followed international ethic requirements for human tissues. It was submitted to the Ethics Committee of the French Society of Ophthalmology (IRB 00008855 Société Française d’Ophtalmologie IRB#1) who waived approval for this type of study. Donor tissue procurement fulfilled all the French legal requirements including absence of the donor in the French National Registry of Opposition to donation and positive family testimony.

### Donor Corneal Tissue

Corneoscleral rims were obtained during surgery after 8-mm trephination of the graft. Donor corneas were obtained from the EFS - Ile-de-France cornea bank (Paris, France). The central part of the donor cornea was transplanted to the scheduled recipient and the remaining corneoscleral rim was used for cell culture. 

### Preparation of Explants

Superficial limbal explants were prepared under a laminar flow. A stromal dissection between the anterior and the mid stroma was carried out using a 15° blade and the sclera was carefully removed with scissors resulting in superficial limbal rims [33]. Six explants with homogeneous length (4 mm) were obtained from each limbal rim using scissors.

### Culture Media

Limbal explants were cultured in cholera toxin-free Green medium [34]. The medium was composed of a 3:1 mixture of calcium-free Dulbecco’s Modified Eagle’s Medium (Dutscher, Brumath, France) and Ham F12 medium (Invitrogen, Cergy Pontoise, France) with 10% fetal bovine serum (Invitrogen), 1 mM/ml HEPES buffer (Invitrogen), 5 µg/ml human recombinant insulin (Actrapid^®^, Novo Nordisk, Paris, France), 0.4 µg/ml hydrocortisone (Pharmacia, Pfizer, Paris, France), 4 µM/ml L-glutamine (Invitrogen), 2 pM/ml tri-iodo thyronine (Sigma, Saint-Quentin en Yvelines, France), 200 nM/ml adenine (Sigma), 100 IU/ml penicillin (Invitrogen), 100 µg/ml streptomycin (Invitrogen), 0.25 µg/ml amphotericin B (Invitrogen), 2 µg/ml isoproterenol (Hospira, Asnières, France) and 10 ng/ml human recombinant Epithelial Growth Factor (EGF) (Sigma). 

### Cell Culture

**Figure 1:**
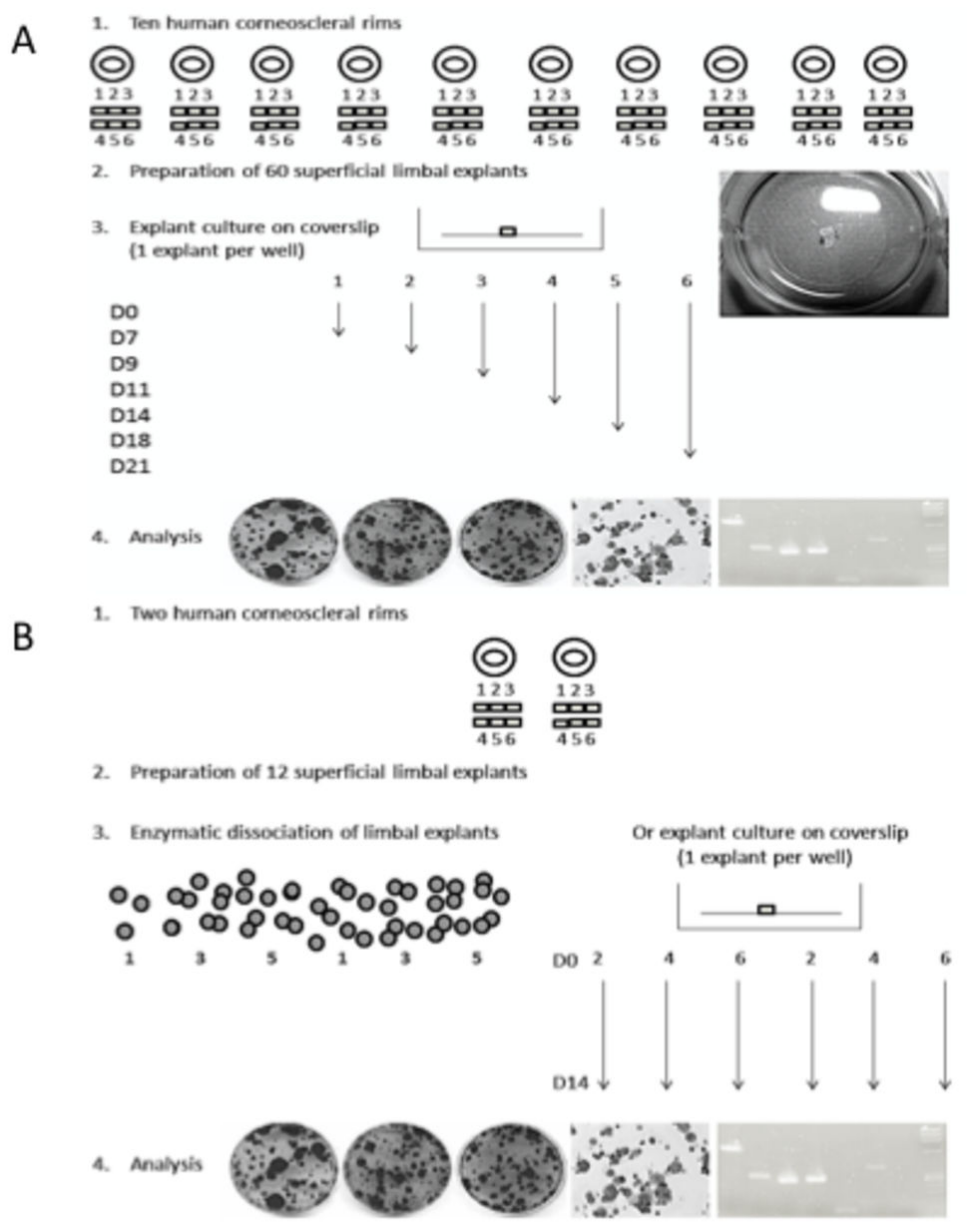
Experimental design. **A**. First series of experiments: 60 human superficial limbal explants were sutured on a tissue-culture treated round coverslip epithelium side up and cultured for 7 to 21 days using 6-well plates. **B**. Second series of experiments: 6 superficial limbal explants from two human donor corneas (3 per cornea) were sutured and cultured for 14 days and the remaining 6 explants were digested with collagenase A.

In a first series of experiments (Figure 1A), the 60 superficial limbal explants from ten human corneas were sutured on a tissue-culture treated round coverslip (320mm^2^, Thermanox, Nunc; one explant per coverslip) epithelium side up and cultured using 6-well plates (907 mm^2^; Becton Dickinson, France). Under laminar flow, one interrupted suture (Vicryl 7-0, Ethicon, Issy-les-Moulineaux, France) was placed to maintain the explant attached to the coverslip. Surgical instruments were used to suture through the coverslip. The coverslip was then put in a well and covered with 2 ml of medium. The medium was changed three times a week. The six explants from each cornea were cultured for 7, 9, 11, 14, 18, and 21 days at 37°C with 5% CO_2_. In a second series of experiments (Figure 1 B), six superficial limbal explants from two human donor cornea (3 per cornea) were sutured and cultured for 14 days and the remaining six explants were digested using 1mg/ml collagenase A ( Roche Diagnostics, Mannheim, Germany) in cholera toxin-free Green medium at 37°C for 18 hours . Cells were collected from the incubated tissues and dissociated into single cells by pipetting.

### Growth assay

At the end of culture and after enzymatic dissociation and trypan blue staining, living cells grown out of the explants were counted. Growth kinetics was expressed as the mean number of trypan blue negative cells according to culture time (n = 10 for each culture time point).

### Colony Formation Assay

The clonal growth ability of cultured limbal epithelial cells was evaluated by determining colony-forming efficiency (CFE). Swiss albino murine 3T3 fibroblasts (ATCC, Molsheim, France) were treated with 4µg/ml mitomycin C for 2 hours and then trypsinized and plated at a density of 2x10^4^/cm^2^ onto six-well culture plates. Cultured cells and enzymatically dissociated cells were seeded at low density (1000 cells/well) in six-well culture plates on 3T3 fibroblasts feeder layers [12]. Cultures were incubated at 37°C under 5% CO_2_. They were observed three times a week by inverted phase-contrast microscopy. Growing epithelial cells were easily differentiated from feeder cells by morphology (i.e., polygonal versus spindle-shaped cells). A colony was defined as a group of eight or more contiguous adherent epithelial cells as described elsewhere [35-37]. Given the low density of seeded epithelial cells, colonies were not contiguous. The epithelial colonies were fixed on day 12 and stained with crystal violet and photographed or dissociated enzymatically and concentrated by cytospin for immunocytochemistry. The CFE was defined either as the percentage or the absolute number of cells forming colonies as follows: CFE (%) = (number of counted colonies / number of seeded cells) x 100, CFE (N) = CFE (%) x number of living cells at the end of primary culture.

### Immunocytochemistry

Cultured cells were enzymatically dissociated and concentrated by cytospin. After washing in PBS, cells were fixed for 10 mins with 4% paraformaldehyde and incubated for 30 mins in PBS containing 1% bovine serum albumin (BSA) and 0.3% Triton X 100 to permeabilize cells and to block non-specific staining. The endogenous peroxidases were quenched with 0.3% H_2_O_2_ during 10 mins. Cells were incubated for 30 mins at room temperature with primary antibodies against cytokeratin 3 1:200 (Clone AE-5; Dako Trappes, France), cytokeratins 4, 5, 6, 8, 10, 13, and 18 1:100 (MNF116; Dako), cytokeratin 19 1:50 (clone BA17, Dako), vimentin 1:200 (clone V9, Dako), p63 1:50 (clone 4A4, Dako), and p63 α (Cell Signaling Technologie, Danvers, USA) followed by incubation with the biotinylated secondary antibody using a LSAB2 system-HRP Kit (Dako) according to the manufacturer’s instructions. DAB was used as peroxidase substrate and specimens were counterstained with haematoxylin.

**Figure 2:**
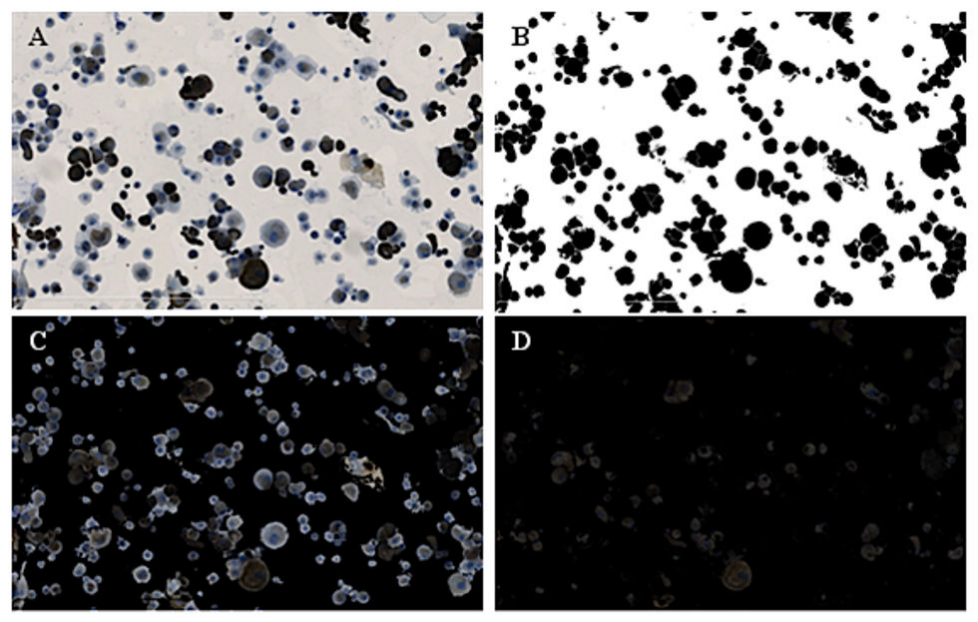
Image analysis of stained cultured cells. An image analysis algorithm was developed to detect individual cell contours and to measure the level of brown coloration using ImageJ. **A**. Primary Image. B. Mask of cells obtained from the green channel of the primary image (Threshold followed by watershed). **C**. Inverted mask of cells redirected to the primary image. **D**. Blue channel of the primary image permitting the brown coloration level to be determined.

Light microscopic images of stained preparations were scanned with the Hamamatsu Nanozoomer Digital Pathology (NDP) 2.0 HT (Hammamatsu Photonics, Massy, France). Five images of each preparation (1 central and 4 peripheral) including at least 500 cells were analyzed with the ImageJ 1.46Rr software (National Institutes of Health, USA). An automated process was developed to measure the intensity of staining (Figure 2). It consists of the following two macros: Macro #1: Channels of the primary image (Figure 2A) are split. Masks of cells are obtained from the green channel of the primary image using threshold followed by watershed (Figure 2B). The mask is inverted, copied, pasted as transparent white, and redirected to the primary image resulting in a secondary image (Figure 2C). Macro #2: Channels of the secondary image are split. The blue channel of the image is converted to mask, inverted, copied, pasted as transparent white, and redirected to the secondary image resulting in the final image that consists of just contours of cells and brown coloration (Figure 2D). The mean level of brown coloration is measured in individual cells. Negative controls were used to determine the threshold of positive staining. This threshold was set as the 90^th^ percentile. The percentage of stained cells was calculated in each preparation. The whole process is fully automated and independent from observer. 

### RNA Extraction, Reverse Transcription, and Quantitative Polymerase Chain Reaction (PCR)

Total RNA was isolated from primary cultured human limbal epithelial cells, from enzymatically dissociated cells, and from clones using the MagNA Pure Compact RNA isolation Kit (Roche Diagnostics, Germany) according to the manufacturer’s instructions. The amount of total RNA isolation was quantified by optical density at 260 nm. cDNA was reverse-transcribed from 1 to 2 mg of total RNA by high capacity cDNA transcription kit (Applied Biosystems; Villebon-sur-Yvette, France). Quantitative polymerase chain reaction (qPCR) amplification of different genes was carried out in a 25 mL solution containing cDNA, Solaris Gene Expression Assay Mix, and Maxima Probe qPCR Master Mix (Thermo Fischer; Fermantas, France). The sequences of the probes used for the qPCR are listed in Table 1. The results of quantitative real time PCR were analyzed by the comparative threshold cycle method and normalized by β-actin as an internal control. The β-actin gene was used as an endogenous reference for each reaction to correct differences in the amount of total cDNA added. Relative change in gene expression was analyzed by the comparative threshold cycle (Ct) method using results obtained for each gene for dissociated cells as a reference. To normalize the amount of target gene in each sample, the difference in Ct (ΔCt) was calculated by subtracting the average Ct of β-actin from that of each gene. The amount of relative target gene mRNA was expressed relative to the amount present in the reference using the following formula: 2^- ΔΔCt.^

**Table 1:** Probes Sequences.

**Genes**	**Probe Sequences**		
CK3	P: CTCCAGCAGCAGGGCAC		
Vimentin	P: TGCGTGAAATGGAAGAG		
Ck19	P: GTGCCACCATTGAGAAC		
ABCG2	P: ATAGCTCAGATCATTGTCCA		
DeltaNp63 α	P :CGAAGCGCCCGTTTCGTCAGAACAC		
ACTB	P: ACCGCGAGAAGATGACC		

### Statistical Analysis

One-way ANOVA was used to compare continuous variables between groups. The Brown–Forsythe test was used to examine homogeneity of variance between groups. Where a significant difference between groups was detected, a post-hoc analysis was performed with the Tukey post-hoc test where group variance was homogeneous and the Games–Howell post-hoc test where group variance was not homogeneous. 

## Results

### Donor Tissue

Twelve human donor corneas were used in this study. The average donor age was 67+10 years (SD, range 52 to 77 years). The time from death to tissue procurement ranged between 3 and 27 hours (mean 17+8 hours). All corneas had been organ-cultured as previously described [34,35] for an average of 11 ± 10 days before trephination.

### Growth Kinetics

**Figure 3:**
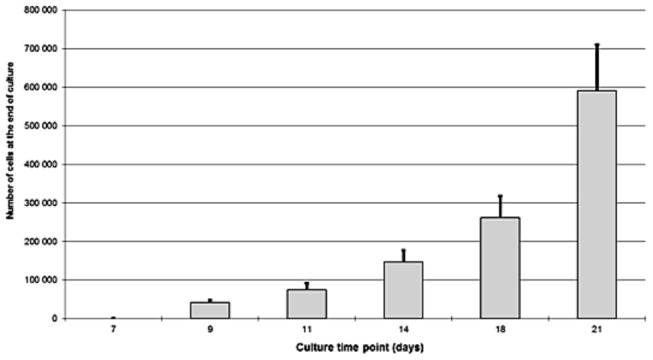
Number of cells after human limbal explant culture. Ten limbal rims retrieved from 10 human corneas were divided in 6 explants each and cultured for 7, 9, 11, 14, 18, or 21 days. Only trypan blue negative cells grown out of the explants were counted.

After 7 days of culture no cell growth could be detected by means of inverted light microscopy. Figure 3 shows growth kinetics from day 9 to day 21.

### Clonal Growth and Phenotypic Characterization

**Figure 4:**
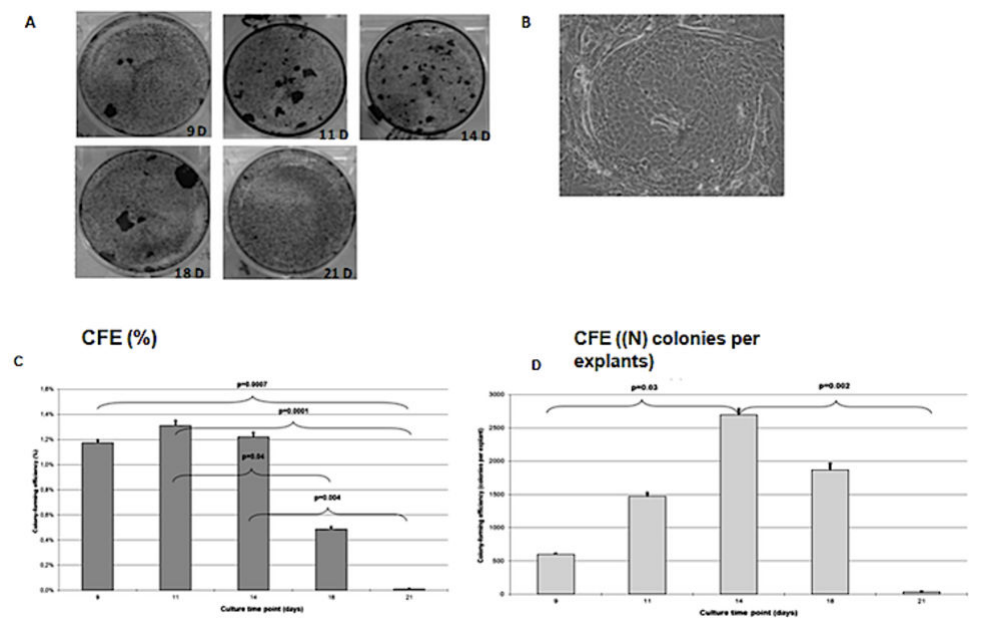
Clonal growth on a 3T3 feeder cell layer and colony-forming efficiency. **A** Macroscopic appearance of colonies obtained from cells grown from explants cultured for 9, 11, 14, 18, and 21 days and stained with crystal violet. **B** Photograph of microscopic appearance of limbal stem cell (LSC) colonies. At the end of primary culture, cells were dissociated and cultured with mitomycin-arrested 3T3 murine feeder cells for 12 days (2 cultures for each primary culture). Colonies were assessed at the end of culture. **C** Significantly higher CFE (%) was obtained after 9, 11, and 14 days of primary culture compared with 21 days (p=0.00002; ANOVA). Shown are mean + standard errors of the mean and post-hoc tests. **D** The primary culture time significantly influenced the CFE (N) (number of cells obtained at the end of primary culture * number of clones / number of seeded cells). Significantly higher CFE (N) was obtained after 14 days of primary culture compared with 9 and 21 days. (p=0.004; ANOVA). Shown are mean + standard errors of the mean and post-hoc tests.

In the first series of experiments ten human limbal rims were divided in six explants each and cultured for 7, 9, 11, 14, 18, and 21 days. After primary culture, cells were dissociated and cultured with mitomycin-arrested 3T3 murine feeders for 12 days. Colonies were obtained after 9, 11, 14, and 18 days of culture but not at D21. Colonies had a smaller size at D14 than at D11 and D18 (Figure 4A). CFE (%) was high at D9, D11, and D14, with no significant differences (p > 0.97) between these three time points, and it significantly decreased at D18 and D21 (p < 0.05) (Figure 4C). 

When the number of colonies per explants was compared, significantly higher CFE (N) was obtained after 14 days of primary culture than after 9 and 21 days (p=0.004) (Figure 4D). 

**Figure 5:**
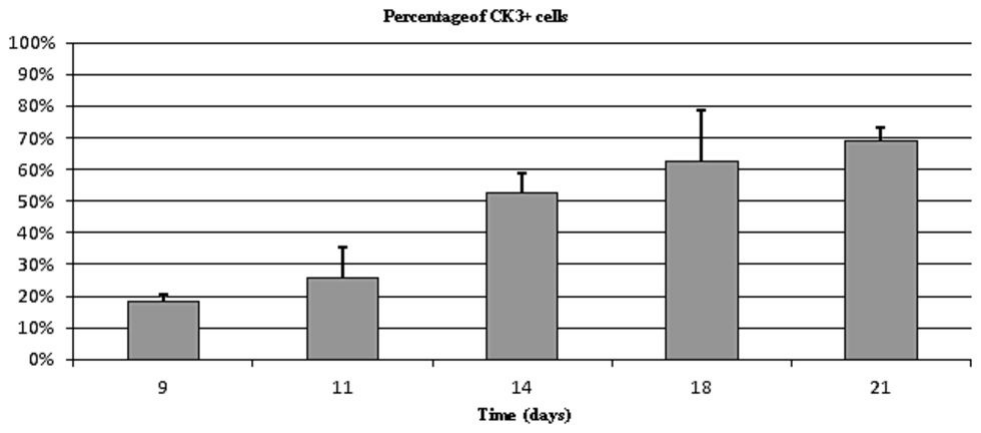
Percentage of CK3-expressing cells after primary culture of human limbal explants for 9 to 21 days. CK3 expression increased with culture time from day 9 to day 21 (p= 0.009).

Immunocytochemical analysis of the cells at the end of primary culture and the cells dissociated from the clones [staining with antibodies against suggested limbal progenitor markers (p63, p63α) and differentiation markers (CK3, MNF116, CK19, and vimentin)] revealed the presence of differentiated cells from day 9 to day 21 in the primary cultures (mean percentage of positive cells ± standard error of the mean: CK3, 45.7±6.3%; MNF116, 69.0±6.3%; CK19, 59.2±6.9%; vimentin, 42.9±7.8%), with significant (p = 0.009) increase for CK3 from D9 to D21 (Figure 5) and no significant variations according to culture time for other differentiation markers (p > 0.05). It showed a steady rate of differentiated cells in the clones obtained after each primary culture time point (CK3, 18.0±4.8%; MNF116, 34.4±9.6%; CK19, 30.5±6.9%; vimentin: 21.4±7.7%). Positive nuclear p63 immunostaining was observed both in primary cultures (16.4±9.9%) and in clones (8.7±4.3%) with no significant variations according to culture time (p > 0.05). p63α, which has been reported to be a specific isoform and marker for progenitor identification [36,37] was expressed both in the primary cultures (16.4±9.9%) and in the clones (7.9±4.3%) with no significant variations according to culture time (p > 0.05).

Quantitative real time PCR showed transcript expression for CK3 (primary cultures, 0.26±0.21; clones, 0.07±0.02), CK 19 (primary cultures, 7.7±2.0; clones, 14.8±9.6), vimentin (primary cultures, 0.10±0.02; clones, 0.09±0.03), and Delta N p63α (primary cultures, 0.15±0.08; clones, 0.12±0.04). 

**Figure 6:**
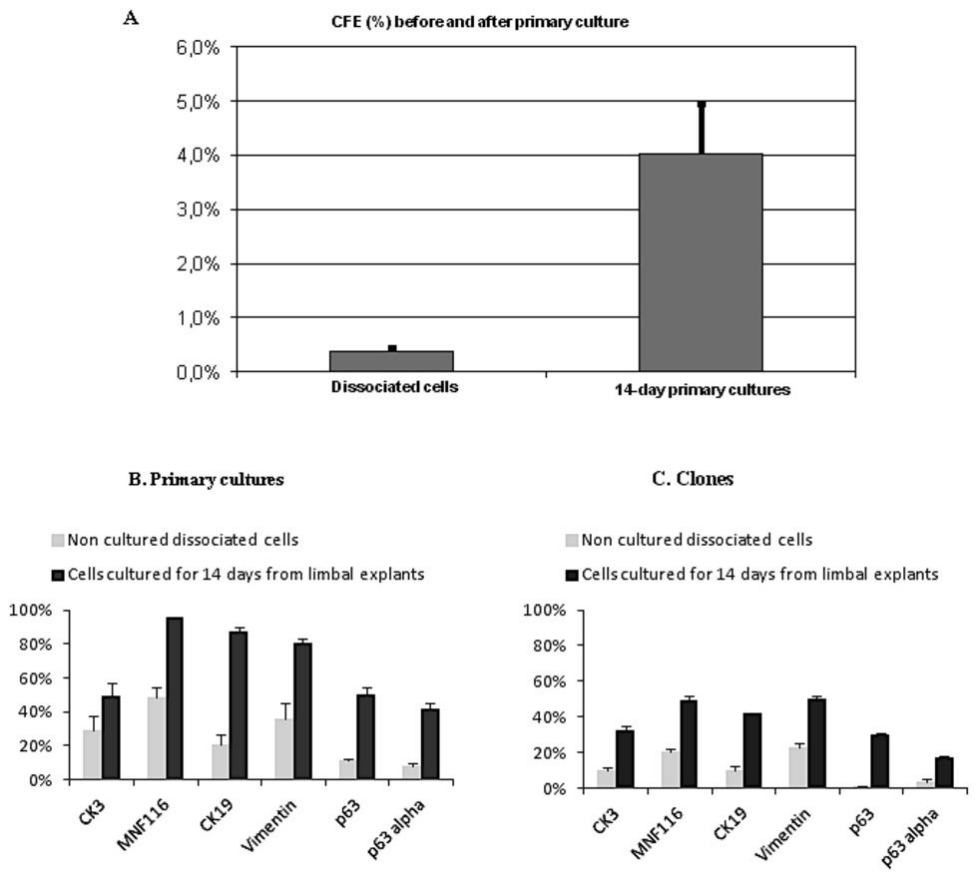
Comparison of dissociated limbal epithelial cells with cultured limbal epithelial cells for 14 days. **A** Colony forming efficiency expressed as the percentage of seeded cells giving rise to clone (CFE %). Significantly higher CFE (%) (p=0.000002) was obtained after 14 days of primary culture of limbal explants compared with non-cultured dissociated limbal epithelial cells. **B, C** Immunohistochemical analysis of enzymatically dissociated cells and cells cultured for 14 days from human limbal explants. **B** Primary culture: expression of differentiation markers and progenitors markers was significantly higher in cells cultured for 14 days than in dissociated cells from the explants (p < 0.02) except for CK3 (p = 0.44). C Clones: immunostaining of differentiation and progenitor markers was significantly higher in colonies obtained from cells cultured for 14 days than in colonies obtained from cells dissociated from explants (p < 0.02). Shown are mean + standard errors of the mean.

In the second series of experiment, two human limbal rims were divided in six explants each. Three explants per cornea were cultured for 14 days and three were dissociated by collagenase A. Dissociated cells obtained from cultured and non-cultured explants were plated on mitomycin C 3T3 feeder layers to analyze their clonogenic potential. The percentage of seeded cells giving rise to clones was 4.03+0.91% (mean + standard error of the mean) after primary epithelial cell culture for 14 days and 0.36+0.08% with dissociated limbal epithelial cells (p=0.000002) (Figure 6A).

Phenotype analysis of cells dissociated from the explants and cells cultured for 14 days showed a significant (p < 0.02) increase in differentiation markers and progenitor markers in cells cultured for 14 days as compared with dissociated cells from the explants except for CK3 that did not significantly increase (p = 0.44) (Figure 6B). Immunostaining for differentiation and progenitor markers was significantly higher (p < 0.02) in clones obtained from cells cultured for 14 days than in clones obtained from cells dissociated from explants. 

**Figure 7:**
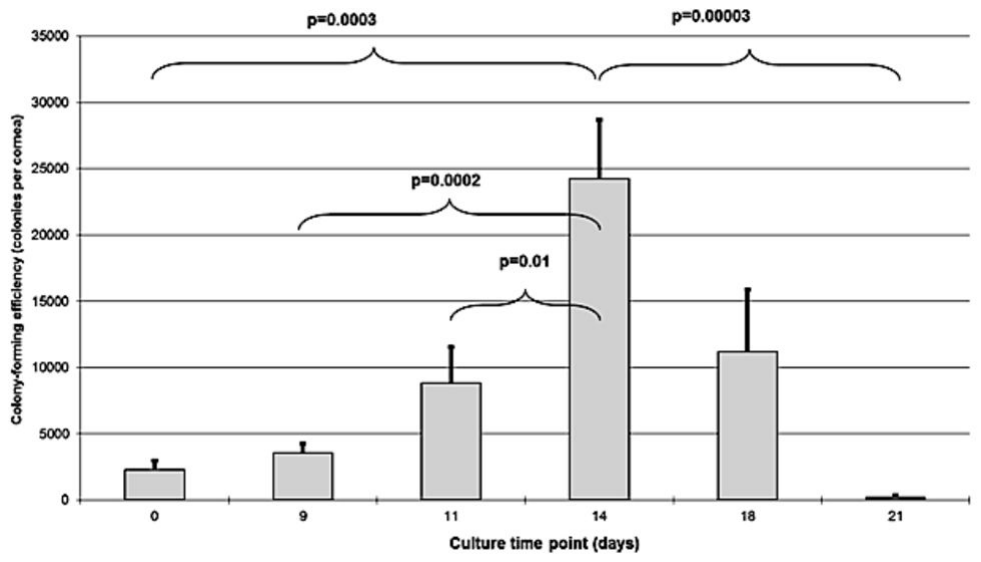
Colony-forming efficiency (N) expressed as the number of cells giving rise to clones per cornea. The number of cells giving rise to clones increased from an average of 2275 per cornea for non-cultured cells to 24266 per cornea for cells obtained after culture of limbal explants for 14 days and decreased afterwards (p<0.00001). This figure was close to 0 after 21 days of primary culture. Shown are mean + standard errors of the mean and post-hoc tests.

The number of cells giving rise to clones per cornea was calculated from CFE (%) and cell counts. It increased from an average of 2275 per cornea for non-cultured cells to 24266 per cornea for cells obtained after culture of limbal explants for 14 days and decreased afterwards (p<0.00001) (Figure 7).

## Discussion

Cultured autologous limbal epithelial cell transplantation is the promising treatment modality for limbal stem cell deficiency with an overall success rate of 76% [29]. Cultures in which p63-bright holoclone-forming cells constituted more than 3% of the total number of clonogenic cells were shown to be associated with successful transplantation in 78% of patients, whereas only 11% of patients obtained stable ocular surface when transplanted with cultures containing less than 3% of stem cells. These results suggest that the success rate of cultured limbal epithelial cell transplantation depends on the quality of donor tissues and the percentage of holoclone-forming limbal stem cells in culture [29,41]. 

Whereas markers such as p63α are useful to easily assess the percentage of progenitors in culture, none of them has been shown to be a specific marker of limbal stem cells. The reference method to make sure that progenitors are present in culture is the widely used Colony Formation Assay [3–5,19,28,35–38,40,42,43]. In this assay limbal epithelial cells are seeded at very low density (1 cell / mm^2^) on a feeder layer of mitomycin-arrested 3T3 fibroblasts. After 12 days of culture the number of colonies is between 10 and 40 per well (i.e., 1 colony per 23 to 90 mm^2^). Each growing colony is assumed to originate from a single original founding cell. Even if more than one cell are seeded in one well and no cloning rings are used it seems unlikely that a colony could originate from several seeded epithelial cells.

In the present study, we found that human limbus contained an average of 2275 cells giving rise to clone. This result is in good agreement with those reported by Di Iorio et al. who found that the number of p63α positive cells was an average 55 per mm [39]. For a cornea with a 12-mm diameter, the total number of limbal p63α positive cells would be 2075.

Our study demonstrates that culture of human limbal explants in a cholera-toxin free defined medium with no feeders permits true expansion of progenitors. A 10-fold expansion of progenitors occurs during the first 2 weeks of culture followed by a decrease and finally a complete loss of progenitors after 3 weeks while cells undergo differentiation with a maximum differentiation observed after 3 weeks. The colony forming efficiency of limbal epithelial progenitor cells increased from 9 to 14 days and then decreased with increasing culture time. The highest number of colonies per explant was observed at D14. The expression level of the corneal epithelial cell differentiation marker (CK3) increased with culture time in primary cultured explants from 9 to 21 days. 

Our findings show that the Clonal growth potential, assessed by clone numbers, declined with time after 14 days of culture. Similar finding was observed by Li W et al. when they cultured explants on intact amniotic membrane [42]. The loss of clonal growth potential in limbal epithelial progenitor cells also occurred in explants cultured on other substrates, such as denuded amniotic membrane and plastic [43]. 

Cultured limbal epithelial cells expressed not only PanCK (Figure 4), confirming their epithelial status, but also p63 and p63α, indicating that they originated from the limbal basal epithelial progenitor cells [44-46].

The percentage of colonies obtained after culture of the limbal explants for 14 days was significantly higher than that obtained with dissociated limbal epithelial cells showing that true expansion of limbal epithelial progenitors occurred during explant culture (Figure 6A). Our results provide evidence that during the first two weeks of culture, expansion of progenitors occurs together with early cell differentiation towards corneal epithelial cell phenotype. Afterwards progenitor expansion is no longer observed and cultured cells are mainly differentiated.

## Conclusion

In conclusion, culture of explants in a feeder-free system led to the expansion and preservation of progenitor cells after 14 days of culture. The number of progenitor cells decreased and the number of differentiated cells increased when the culture time exceeded 2 weeks. This system allows high number of progenitor cells to be obtained with no feeders which is an important factor for transplantation.
